# Identifying a Molecular Mechanism That Imparts Species-Specific Toxicity to YoeB Toxins

**DOI:** 10.3389/fmicb.2020.00959

**Published:** 2020-05-21

**Authors:** Jessica R. Ames, Julia McGillick, Tamiko Murphy, Eswar Reddem, Christina R. Bourne

**Affiliations:** Department of Chemistry and Biochemistry, University of Oklahoma, Norman, OK, United States

**Keywords:** YoeB toxin, toxicity, *E. coli*, *A. tumefaciens*, ribosome-dependent mRNase, species-specific toxicity

## Abstract

The ribosome-dependent *E. coli* (Ec) mRNase toxin YoeB has been demonstrated to protect cells during thermal stress. *Agrobacterium tumefaciens* (At), a plant pathogen, also encodes a YoeB toxin. Initial studies indicated that AtYoeB does not impact the growth of Ec, but its expression is toxic to the native host At. The current work examines this species-specific effect. We establish the highly similar structure and function of Ec and AtYoeB toxins, including the ability of the AtYoeB toxin to inhibit Ec ribosomes *in vitro*. Comparison of YoeB sequences and structures highlights a four-residue helix between β-strands 2 and 3 that interacts with mRNA bases within the ribosome. This helix sequence is varied among YoeB toxins, and this variation correlates with bacterial classes of proteobacteria. When the four amino acid sequence of this helix is transplanted from EcYoeB onto AtYoeB, the resulting chimera gains toxicity to Ec cells and lessens toxicity to At cells. The reverse is also true, such that EcYoeB with the AtYoeB helix sequence is less toxic to Ec and gains toxicity to At cultures. We suggest this helix sequence directs mRNA sequence-specific degradation, which varies among proteobacterial classes, and thus controls growth inhibition and YoeB toxicity.

## Introduction

Toxin-antitoxin (TA) systems are an important mode of intracellular prokaryotic and archaeal regulation (Pandey and Gerdes, 2005; Unterholzner et al., 2013; Hall et al., 2017; Harms et al., 2018; Ronneau and Helaine, 2019). TA systems are widespread and found encoded on phage islands (Lehnherr et al., 1993), plasmids (Ogura and Hiraga, 1983; Gerdes et al., 1986), and chromosomes (Pandey and Gerdes, 2005). Type II TA systems are the best understood, and contain both a protein toxin and a more labile protein antitoxin (Kedzierska and Hayes, 2016; Harms et al., 2018). The role of the antitoxin is twofold: firstly, to neutralize the toxin, and, secondly, to serve as a self-repressor for the transcription of its operon (Hayes and Kedzierska, 2014; Muthuramalingam et al., 2016). Cellular proteases, such as Lon and Clp, then degrade the antitoxin (Tsuchimoto and Ohtsubo, 1993; Van Melderen et al., 1994; Lehnherr and Yarmolinsky, 1995; Brzozowska and Zielenkiewicz, 2013; Hayes and Kedzierska, 2014; Muthuramalingam et al., 2016; Page and Peti, 2016). This loss of antitoxin triggers increased transcription and increased availability of intracellular toxin (Donegan et al., 2010; Brzozowska and Zielenkiewicz, 2013). Many toxins have been identified that degrade RNA, including ribosome-dependent RNase toxins in the RelE family (Christensen and Gerdes, 2003; Pedersen et al., 2003) and sub-members YafQ (Prysak et al., 2009; Maehigashi et al., 2015), HigB (Hurley and Woychik, 2009; Schureck et al., 2016), and YoeB (Kamada and Hanaoka, 2005; Yoshizumi et al., 2009; Zhang and Inouye, 2009; Feng et al., 2013; Pavelich et al., 2019).

While plasmid-encoded TA systems have been linked to post-segregational killing (Gerdes et al., 1986) and abortive infection (Dy et al., 2014), the role(s) of chromosomally-encoded TA systems remains controversial (Harms et al., 2018; Fraikin et al., 2020). Potential functions, which are not mutually exclusive nor necessarily shared among all TA systems, include mediating responses to specific stresses, altruistic cell death, or protection from invading genetic material (Magnuson, 2007; Van Melderen and Saavedra De Bast, 2009; Van Melderen, 2010). The chromosomally-encoded YoeB toxin from *E. coli* has been clearly established to mediate ribosome-dependent mRNA cleavage in response to thermal stress (Cherny et al., 2005; Kamada and Hanaoka, 2005; Zhang and Inouye, 2009; Chan et al., 2011; Feng et al., 2013; Janssen et al., 2015; Pavelich et al., 2019). *Candidatus* bacterial strains are endosymbionts with root-associated mycorrhizal fungi, and were noted to contain a chromosomal YoeB-YefM system that was negatively correlated with active bacterial growth and demonstrated toxicity when expressed in an *E. coli* host (Salvioli di Fossalunga et al., 2017). Gram-positive bacteria also encode YoeB-YefM TA systems. In *Streptococcus pneumoniae*, a chromosomal YoeB module is toxic when expressed in *E. coli* and has been associated with tolerance to oxidative stress and contributions to biofilm formation in its native host (Nieto et al., 2007; Chan et al., 2018). This toxicity is similar to results obtained with the highly similar YoeB-YefM system from *Streptococcus suis* (Zheng et al., 2015). Two chromosomal YoeB toxin homologs from *Staphylococcus aureus* have been characterized as having analogous toxicity and *in vivo* functionality as that from *E. coli* (Yoshizumi et al., 2009). *Staphylococcus equorum* SE3 has two YoeB toxins characterized as toxic when overexpressed in *E. coli* cultures (Nolle et al., 2013), as has a YoeB toxin native to *Streptomyces* sp. SCSIO 02999 (Zhan et al., 2019).

In contrast to these previous studies, we find that the chromosomal YoeB toxin from *Agrobacterium tumefaciens* is not toxic to *E. coli*, yet is potentially toxic to its native host. Previous studies on TA systems in *A. tumefaciens* have focused only on tumor-inducing plasmid-borne TA systems (Davis et al., 1992; Wozniak and Waldor, 2009; Yamamoto et al., 2009; Diaz-Orejas et al., 2017). The current work explores a proposed Rel-type toxin from *A. tumefaciens* that is encoded on the essential circular chromosome (Goodner et al., 2001). We demonstrate that this toxin shares sequence and structural similarities, as well as functional activities, with *E coli* ribonuclease YoeB toxin. This toxin from *A. tumefaciens* is able to inhibit translation by ribosomes from *E. coli*, yet it exhibits no toxicity to *E. coli* cultures. It is, however, toxic when expressed in its host organism *A. tumefaciens.* We identified a four amino acid helical segment that varies in sequence between these two species and created chimeric versions in which the helices were exchanged. We demonstrate that the chimeric versions of these YoeB toxins have partially swapped toxicity, validating this short four amino acid helix located between β-strands 2 and 3 of YoeB as a specific toxicity-determining region. Analysis of YoeB sequences further reveals that the amino acid identities at this helix are correlated with different bacterial classes of the proteobacterial Phyla. Overall, these studies highlight how small changes in an overall conserved Type II TA system can result in different host toxicities.

## Materials and Methods

Protein sequence alignments were carried out using UCSF Chimera, and prepared as a figure using ESpript 3 (Robert and Gouet, 2014). All protein structure figures and analysis of contacts were made using UCSF Chimera (Pettersen et al., 2004).

### Cloning, Expression, and Purification

**FIGURE 1:**
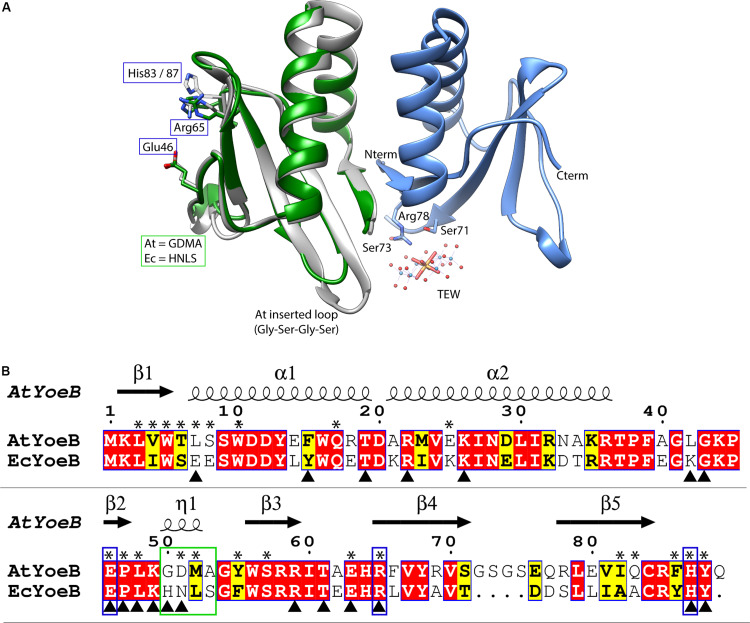
AtYoeB is similar to EcYoeB both in structure and in sequence. **(A)** An AtYoeB dimer (blue and gray ribbons) is found in the asymmetric unit of the crystal, with important crystal packing contacts mediated by a tungstate-terillium compound (TEW). The catalytic residues Glu46, Arg65, and a C-terminal His are conserved (shown in stick form, EcYoeB, (PDB ID 2A6Q) (Kamada and Hanaoka, 2005), shown as green ribbon). **(B)** The sequence of AtYoeB and EcYoeB are 76% similar, depicted with conserved residues in red boxes and similar in yellow boxes. Secondary structure is depicted above the sequence (including 3_10_ helices, indicated as “η”). Blue boxes indicate the catalytic residues, which are conserved, while the green box indicates the specificity-determining helix (determined in the current work). Asterisks denote amino acids contacting the YefM antitoxin; triangles denote contacts with the ribosome (deduced from PDB ID 6OXA) (Pavelich et al., 2019). Antitoxin contacts deduced from superposition of the AtYoeB structure (PDB ID 6N90) onto the *E. coli* YoeB-YefM complex (PDB ID 2A6Q) (Kamada and Hanaoka, 2005).

The AtYoeB and AtYefM genes were amplified from genomic DNA. The YoeB toxin was cloned into a modified pET-28(a) vector containing a C-terminal GST fusion affinity tag in addition to a 6× His tag, while the YefM antitoxin was cloned into the pET15b vector. These were also cloned into the pET-Duet vector, with the AtYefM protein placed in the first multiple cloning site with an N-terminal 6 × His tag, while the second multiple cloning site contained untagged AtYoeB. AtYoeB and EcYoeB were cloned into the pSRK vector (provided by S. Crosson) using a Gibson assembly (NEB) approach. Site-directed mutagenesis (Q5, NEB) was used to generate YoeB toxins with chimeric sequences at the designated helix (e.g., see Figure 1, primers given in Supplementary Table S1). Each construct was verified by Sanger sequencing (see Supplementary Table S1 for sequences, strains, and plasmids).

BL21 (DE3) *E. coli* were transformed with these expression clones and propagated in Lysogeny broth (Miller, Difco) at 37°C, 200 rpm, and induced with 0.5–1 mM isopropyl β-D-1-thiogalactopyranoside (IPTG) when the OD_600_ measured ∼0.6. The temperature was decreased to 16°C for an overnight induction for AtYoeB and the AtYefM-YoeB co-expression, and 4–6 h for AtYefM antitoxin. Harvested cultures were resuspended in 50 mM Tris pH 8.5, 300 mM NaCl, mechanically lysed using an EmulsiFlex-C3 (Avestin), and purified using a Roche HisTrap NiNTA column. Fractions containing AtYoeB-GST were desalted into 50 mM Tris pH 7.5, 150 mM NaCl and incubated overnight at 4°C with 2 U mg^–1^ HRV3c PreScission protease (Sigma), 1 mM DTT, and 1 mM EDTA. Cleavage of the AtYefM 6 × His tag affinity tag similarly was achieved by desalting and incubation overnight at 4°C with 2 U mg^–1^ of thrombin (Sigma) and 2 mM CaCl_2_. The final purification step for each sample, including the co-expressed AtYefM-YoeB, utilized a Superdex 75 10/300 GL column (GE Healthcare) equilibrated in 50 mM Tris pH 8, 150 mM NaCl; protein purity was assessed by electrophoresis using 12 or 18% tris-tricine gels.

### Structure Determination of AtYoeB

Crystallization screening was carried out at 293 K in a 96-well plate using sitting drop vapor diffusion crystallography with the aid of a Mosquito crystallization robot (TPP LabTech). Diffraction quality crystals obtained after 48 hrs with a maximum size of 150 × 150 × 75 μm in a condition of 5% v/v 2-Methyl-2,4-pentanediol (MPD), 10% PEG 6,000, 100 mM 4-(2-hydroxyethyl)-1-piperazineethanesulfonic acid (HEPES) pH 7.5, and 1 mM TEW. Prior to data collection, AtYoeB crystals were briefly rinsed in 30% MPD in the mother liquor and plunged in liquid nitrogen. X-ray diffraction data (extending to 1.75 Å) were collected at 100 K at the SSRL ID14-1 beamline (Stanford, United States) with using an Eiger X 16M detector (Dextris AG). Diffraction data were processed with the program XDS (Kabsch, 2010) and scaled using AIMLESS (Evans, 2006) from the CCP4 software suite (Collaborative Computational Project, Number 4, 1994). Molecular replacement was performed with PHASER (McCoy et al., 2007) using PDB entry 2A6Q (Kamada and Hanaoka, 2005) as a search model. Manual rebuilding of the structure using COOT (Emsley et al., 2010) was alternated with refinement using Phenix (Afonine et al., 2012). TEW was included after inspection of the initial electron density maps during the final stage of model building and refinement. Statistics for data collection and refinement are presented in Supplementary Table S2.

### Evaluation of *in vivo* Toxicity

Plasmids were introduced into bacterial cells by electroporation following manufacturer instructions (Bio-Rad). Cultures of *E. coli* MG1655 were propagated in LB broth at 37°C with shaking, and the pSRK plasmid was selected for by the addition of Kanamycin to 50 μg/mL. Likewise, cultures of *A. tumefaciens* C58 in were grown in MG/L broth at 30°C with Kanamycin at 150 μg/mL for liquid cultures and 300 μg/mL for plated media. Cultures were propagated overnight (16 hr) in the presence of 1% glucose to ensure repression of transcription. These were then inoculated into 96-well plates at a 1:100 ratio containing LB or MG/L broth and Kanamycin, as appropriate, with increasing concentrations of inducer (IPTG). Plates were sealed and incubated in a BioTek plate reader with temperature control and shaking, and the optical density was recorded every 10 min. At desired time points after induction, cultures were sampled by removing 2 μL and serially diluting this in growth media containing selection antibiotic and 0.5% glucose, followed by pipetting dilutions onto selective plated media. These agar plates were then incubated at 30°C for At and 37°C for Ec for 16–20 h, and imaged on a ChemiDoc unit (Bio-Rad).

### Differential Scanning Fluorimetry Assay

Purified protein samples in 50 mM Tris pH 8, 150 mM NaCl were diluted to 36 μM and an equal volume mixed with 5 × SYPRO orange (Invitrogen) to yield 20 μL reactions. Assays were carried out in white 96-well PCR plates using a Roche LightCycler 480II set to the minimum ramp rate from 20 to 95°C with 1 measurement per degree. Resulting melt curves were transformed to the negative first derivative (-d/dT) using manufacturer’s software (StepOne v2.1), visually inspected to reveal the maximum fluorescence change, and replotted using GraphPad Prism (v6.0d). Measurements were repeated three times with essentially identical results, and omission of protein from the samples yielded no change in fluorescence.

### Multi-Angle Light Scattering (MALS) Assay

Purified samples of AtYoeB, AtYefM, and co-expressed AtYeoB-YefM were analyzed for absolute molecular weight determination using a Wyatt miniDAWN Treos system integrated with size exclusion chromatography and a refractive index detector, all maintained at room temperature. The sizing column, a Superdex 200 Increase 10/30 GL (GE Healthcare), was equilibrated in 25 mM HEPES pH 7.5, 150 mM NaCl buffer. The resulting light scattering profiles were analyzed using the ASTRA software (v 6.1, Wyatt Technologies) following manufacturer’s recommendations; concentrations were determined based on the signal from the refractive index detector, as AtYefM contains no tryptophan amino acids, making concentration calculations from absorbance at 280 nm error prone. The resulting data were ported to GraphPad Prism (v 6.0d) and replotted for graphic presentation.

### Biolayer Interferometry (BLI) Assay

In order to measure the binding between the toxin and antitoxin, an NiNTA pin (ForteBio) was incubated with 125nM His-AtYefM, followed by titration of concentrations of AtYoeB (after removal of the 6 × His tag). All solutions were prepared in a 1 × block buffer consisting of 0.5%BSA, 0.05%Tween-20 in 50 mM Tris-HCL pH 8.5 and 300 mM NaCl. Controls were included with each assay, consisting of loading a non-specific protein (His-dihydrofolate reductase) as well as not loading any protein to the NiNTA pin; neither showed appreciable signals of interaction with AtYoeB, and the “empty” pin signal was used to correct for the baseline. Pins were regenerated before and between runs by incubations in 10 mM glycine pH 1.7 alternated with 1 × block buffer for three cycles of 5 s, followed by recharging in 10 mM NiCl_2_ for 60 s. Data were processed with the ForteBio Octet Data Analysis software using best practices. Sufficiently good fits were obtained using a model for a 1:1 stoichiometry (see Supplementary Figure S5 for individual assays and calculations).

### RNA Synthesis

RNA was synthesized from *Sma*I (NEB) linearized plasmid containing the Firefly luciferase gene under control of a T7 promoter (Promega). Linearized DNA was recovered through a standard phenol:chloroform extraction, followed by a back extraction. Glycogen at 10 μg/mL was used as a carrier during an overnight ethanol precipitation at −20°C as described (Xie et al., 2018). Purified DNA substrate was added to the HiScribe^TM^ T7 High Yield RNA Synthesis kit (NEB), and RNA synthesis was carried out according to manufacturer’s directions. The resulting product was electrophoresed on a 1.2% FlashGel^TM^ RNA cassette (Lonza) at 275 V for 8–10 min to assess purity. RNA was purified via phenol:chloroform extraction and back extraction following DNase I (NEB) treatment. The final product was recovered via ethanol precipitation.

### Ribosome Dependent Nuclease Assay

We measured the activity of AtYoeB in the presence of the ribosome by monitoring the production of Green Fluorescent Protein (GFP) using the PURExpress *In Vitro* Protein Synthesis Kit (NEB). Starting substrates were derived from a clone of GFP in a pET28(a) vector (pET28:GFP was a gift from Matthew Bennett, Addgene plasmid # 60733^1^; RRID:Addgene_60733), and reactions contained a final concentration of either 300 ng of linearized GFP DNA or 7.5 μg of GFP RNA (synthesized as described above). Fluorescence was measured every 15 min for 2 h using an excitation wavelength of 485 nm and an emission wavelength of 528 nm in a Synergy H1 Hybrid Multi-Mode Microplate Reader (BioTek). Data were analyzed by subtracting the background fluorescence arising from a reaction with no DNA or RNA substrate to give a corrected fluorescence. The corrected fluorescence at the 2 h time point was divided by the corrected fluorescence of the positive control (containing only the starting substrate with buffer and no toxin), converted to percentages, and graphed as a function of toxin concentration.

### Bioinformatic Analysis of YoeB Sequence Conservation

Aligned YoeB sequences in the Representative Proteomes 15 (548 sequences) were downloaded from the Pfam database (El-Gebali et al., 2019) and used as input to the online WebLogo v2.8.2 tool (Crooks et al., 2004). The evolutionary history was inferred using the Minimum Evolution method (Rzhetsky and Nei, 1992). The evolutionary distances were computed using the p-distance method (Nei and Kumar, 2000) and are in the units of the number of amino acid differences per site. The ME tree was searched using the Close-Neighbor-Interchange (CNI) algorithm (Nei and Kumar, 2000) at a search level of 1. The Neighbor-joining algorithm (Saitou and Nei, 1987) was used to generate the initial tree. All positions containing gaps and missing data were eliminated. There were a total of 82 positions in the final dataset. Evolutionary analyses were conducted in MEGA7 (Kumar et al., 2016) using an alignment generated by PROMALS (Pei and Grishin, 2007).

## Results

### *Agrobacterium tumefaciens* Encodes a Chromosomal YoeB-YefM Toxin Antitoxin Pair

The genes *Atu2017* (UniProt ID A9CID9) and *Atu2018* (UniProt ID Q7CY23) from *Agrobacterium tumefaciens* strC58 were identified as a potential TA pair by the Rapid Automated Scan for Toxins and Antitoxins in Bacteria (RASTA-Bacteria) (Sevin and Barloy-Hubler, 2007). The toxin-antitoxin database (TADB 2.0) predicted *Atu2017* and *Atu2018* to be members of the Par/Rel toxin superfamily fold and the Ph.D. antitoxin superfamily fold (Xie et al., 2018). These predictions support identification of the toxin as a member of the YoeB family, as the cognate YefM antitoxin is housed within the Ph.D. fold (Arbing et al., 2010).

The structure of the YoeB toxin from *A. tumefaciens*, herein referred to as AtYoeB, was determined at 1.75 Å resolution (see Supplementary Table S2). YoeB toxins conform to a previously characterized RNase fold consisting of two helices packed against a twisted four to five-stranded antiparallel beta sheet (Kamada and Hanaoka, 2005; Pavelich et al., 2019). When compared to the EcYoeB toxin, there is an overall RMSD of 0.6 Å for the core residues with 1.2 Å deviation overall (Figure 1A). As can be deduced from the protein sequence alignment in Figure 1, these two toxins are 57% identical (76% similar) at the amino acid level, including absolute conservation of the EcYoeB catalytic residues noted in previous studies (Figure 1B, blue boxes) (Feng et al., 2013; Pavelich et al., 2019). Other similar residues include C-terminal aromatic amino acids that assist in stabilizing the substrate, such as Phe86 or Tyr88 in AtYoeB, Tyr84 in EcYoeB, and Phe91 in the closely related EcYafQ toxin (Maehigashi et al., 2015). The AtYoeB sequence contains four inserted amino acids, located in a loop between beta-strands 4 and 5 (Figure 1). This loop was disordered in one of the two copies of AtYoeB present in the crystallographic asymmetric unit; in addition, this loop makes interactions with the tungstate-terillium compound that was critical for bridging important crystal contacts and that was required to obtain usable diffraction quality crystals (Figure 1A). This larger loop does not appear to impact the fit of the toxin within the ribosome A site (Supplementary Figure S1A). Regions of basic charge on the surface are conserved, and when superposed with the YoeB structure in the ribosome site the amino acids interacting with the ribosome are also largely conserved (also see Supplementary Figure S1). Of the 35 amino acids differences between EcYoeB and AtYoeB, eleven are located at the antitoxin interaction surface (Figure 1B, asterisks), which is highly specific for individual cognate pairs (Aakre et al., 2015).

The YoeB dimer interface is well conserved among this class of toxin, largely solidified by two tryptophan residues from each monomer (conserved at positions 5 and 10 for both At and EcYoeB) forming an aromatic ring cluster, in addition to polar interactions mediated by conserved Tyr13, Gln17 and Asn18 side chains (Supplementary Figure S1B). Additional interactions are noted for AtYoeB at positions that differ from its Ec counterpart: (Ec to At) Leu14Glu, Glu18Arg, and Lys32Arg (Supplementary Figure S1). These changes generate additional polar interactions for the AtYoeB dimer.

### AtYoeB Is Toxic to *Agrobacterium* but Not *Escherichia* Cultures

The YoeB toxin encoded in Escherichia coli (EcYoeB) requires co-expression with the cognate antitoxin due to its marked inhibition of *E. coli* growth (Cherny et al., 2005; Kamada and Hanaoka, 2005; Zhang and Inouye, 2009; Chan et al., 2011; Aakre et al., 2015; Pavelich et al., 2019). Interestingly, AtYoeB appears much less toxic during over-expression. We have previously measured the toxicity of AtYoeB to *E. coli* (strain BL21 DE3) and noted no impact on cells until after more than 10 h of overexpression (Ames et al., 2019). We are able to produce recombinant AtYoeB in the absence of antitoxin with yields of 3-5 milligrams per liter of culture, and with greater than 7 milligrams per liter of culture when the antitoxin is co-expressed (see Materials and Methods section for induction details).

**FIGURE 2:**
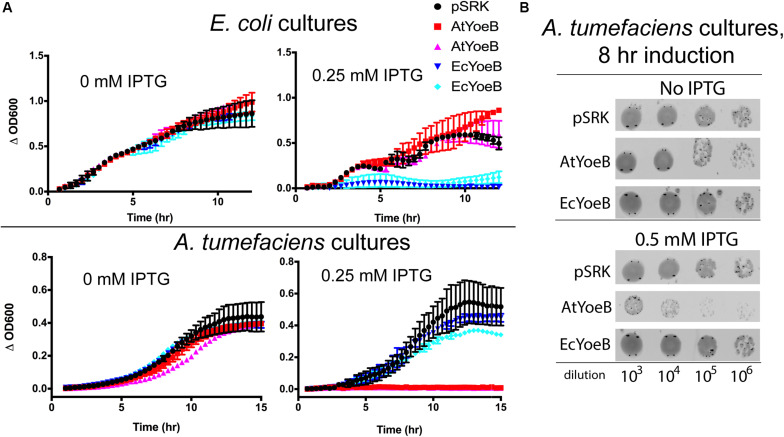
The toxicity of the YoeB toxins is specific for the native host. **(A)** Expression of two independent clones was carried out in both *E. coli* MG1655 and *A. tumefaciens* C58 as a function of inductant (IPTG) and compared to the expression vector with no inserted gene (pSRK). Toxicity is evident for both clones of EcYoeB only in *E. coli*, while toxicity for both AtYoeB clones is only observed in *A. tumefaciens*. The deviation between two technical replicates is plotted as error bars; two independent replicates were carried out (see Supplementary Figures S2, S3 for additional replicate). **(B)** Spot dilutions for each culture demonstrates that the AtYoeB toxin is mediating cell death, not just causing cell dormancy.

We carried out expression studies to quantify the impact of EcYoeB and AtYoeB on each of their respective hosts (Figure 2A), which recapitulated our observations during protein expression. Using two independent clones of the AtYoeB toxin, we observed a total lack of toxicity (e.g., equivalent growth as the control vector with no toxin inserted) to *E. coli* (strain MG1655) up to 2 mM IPTG induction (see Supplementary Figure S2). In contrast, these AtYoeB clones were very toxic to *A. tumefaciens* at IPTG concentrations as low as 0.125–0.25 mM (see Supplementary Figure S3), while the EcYoeB clones demonstrated no toxicity up to 1–2 mM IPTG in *A. tumefaciens*. When these clones were harbored in *E. coli* cells, both the EcYoeB clones demonstrated reduced growth indicative of toxicity at induction levels as low as 0.06 mM IPTG (the lowest concentration tested, see Supplementary Figure S2). Cultures of *A. tumefaciens* were serially diluted and spotted on growth media to assess if the toxicity was bacteriostatic or bactericidal. *A. tumefaciens* cells were not able to re-grow after removal of inductant and re-culture (Figure 2B), indicating a likely bactericidal effect by 8 h post-induction.

### AtYoeB Exhibits Typical Properties of a YoeB Toxin, Including Inhibition of *E. coli* Ribosomes

**FIGURE 3:**
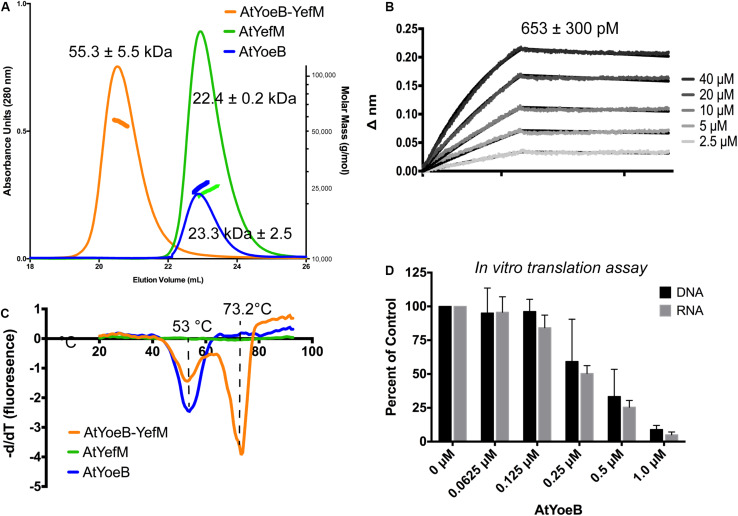
Dimeric AtYoeB interacts tightly with AtYefM, and is functional in inhibiting translation from *E. coli* ribosomes. **(A)** Size-exclusion multiple angle light scattering (SEC-MALS, signal depicted as colored markers along with the chromatograph traces) establishes the dimeric state of the individual partners and the resulting heterotetramer. AtYefM (green) contained the His affinity tag, while AtYoeB (blue) was analyzed after removal of the GST-His affinity tag (*n* = 2 for each sample, see Supplementary Figure S4). **(B)** Biolayer interferometry (BLI) was carried out by capturing His-AtYefM on NiNTA pins and interactions were measured as AtYoeB toxin was titrated (*n* = 3). A one-to-one stoichiometric fit resulted in an interaction strength of 653 pM (see Supplementary Figure S5). **(C)** Differential scanning fluorimetry experiments were used to determine that the AtYefM antitoxin (green) does not display an appreciable melting transition in the absence of the AtYoeB toxin (blue), while the complex (orange) yields a single transition with a stabilizing effect versus the toxin alone (*n* = 3). **(D)** *In vitro* coupled transcription translation assays were carried out using either linearized DNA or *in vitro* transcribed RNA coding for Green Fluorescent Protein and incubated with increasing concentrations of AtYoeB; the resulting fluorescence was measured and normalized to control samples containing no AtYoeB (*n* = 3).

This remarkable species-specific toxicity led us to further test the AtYoeB for canonical properties of this toxin family. The dimeric state of AtYoeB was verified using MALS, which determined an absolute molecular weight of 23.3 kDa (± 2.5, *n* = 2) (Figure 3A). The purified antitoxin, YefM, was also found to be dimeric by MALS (22.4 ± 0.2 kDa, *n* = 2) (Figure 3A). To examine the stoichiometry of the complex, we turned to a co-expression model. Purification relied on the affinity tag of the antitoxin and yielded a single species by size exclusion (Supplementary Figure S4). This species contains the complex of AtYoeB and AtYefM, and by MALS analysis this complex is a 1:1 interaction resulting in a heterotetramer (55.3 ± 5.5 kDa, *n* = 3, Figure 3A).

The dimeric AtYoeB toxin interacts strongly with the cognate AtYefM antitoxin, measured by Biolayer Interferometry, yielding a calculated K_*D*_ of 653 ± 300 pM using a model for a 1:1 fit (Figure 3B and Supplementary Figure S5). Consistent with this tight interaction, the Tm for this complex by Differential Scanning Fluorimetry (DSF) shifts higher to 73.2°C (Figure 3C). The AtYoeB dimer was found to have a relatively stable melting transition (Tm) value by DSF of ≅53°C (Figure 3C). In contrast, the YefM antitoxin has no discernable transition in this assay, indicating that it lacks a hydrophobic core that can undergo denaturation. This is consistent with an extended helical structure seen previously for EcYefM (Kamada and Hanaoka, 2005; Kumar et al., 2008). The complex of AtYoeB-YefM maintains a signal corresponding to toxin denaturation, and in addition gains a species with a stabilized structure with a Tm of 73.2°C, likely correlated with a previously determined role of this TA system in thermal stress responses (Janssen et al., 2015).

Importantly, the AtYoeB toxin is able to inhibit the translation activity of *E. coli* ribosomes (Figure 3D). Translation inhibition arises from YoeB binding the ribosomal A site and proceeding to cleave mRNA (Christensen et al., 2004; Zhang and Inouye, 2009; Schureck et al., 2015). We utilized an *in vitro* (cell-free) coupled transcription-translation reaction to assess the dose-dependent impact of AtYoeB on *E. coli* ribosome translation by measuring the production of green fluorescent protein (Figure 3D). This assay reveals that AtYoeB is able to inhibit *E. coli* ribosomes in a dose-dependent manner, and further, that in this coupled system the inhibition is independent of initiation with DNA or RNA templates.

### The Mechanism for AtYoeB Species-Specific Toxicity Is Encoded in a Short 3_10_ Helix

**FIGURE 4:**
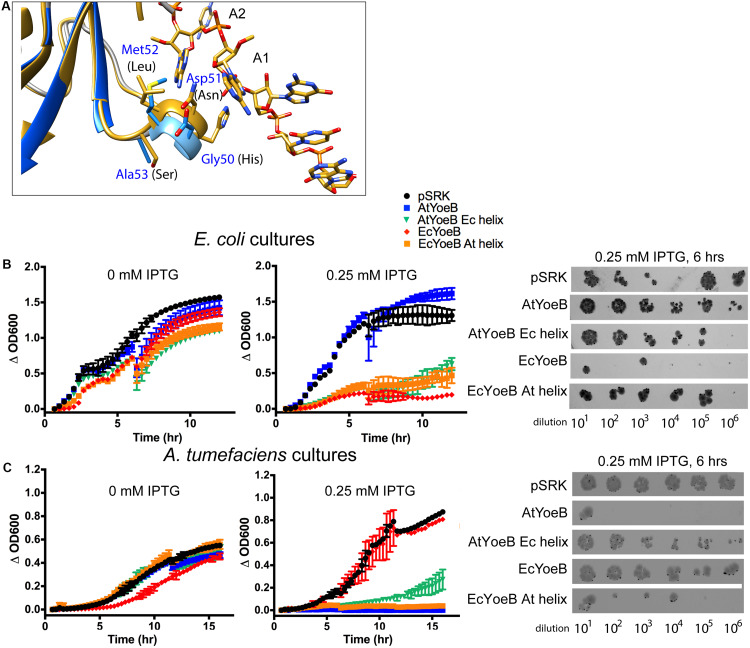
A four amino acid helix imparts species-specific toxicity. **(A)** The AtYoeB structure (PDB 6N90, blue) is superposed onto EcYoeB (PDB 6OXA, yellow) (Pavelich et al., 2019) as positioned within an *E. coli* ribosome. The four amino acids in AtYoeB (GDMA) are labeled in blue, those of EcYoeB (HNLS) are in black. The targeted mRNA is shown as yellow sticks (oxygen atoms, red; nitrogen atoms, blue), and the first base (A1) and second base (A2) of the codon are labeled. **(B,C)** Expression of the wild type toxins imparts toxicity to the native host species (as in Figure 2), however, chimeras (AtYoeB with EcYoeB helix, green; EcYoeB with AtYoeB helix, orange) that have the four amino acid helix sequence swapped are now toxic to the other species. Further, the toxicity of these chimeras to the native species is attenuated. The deviation between two technical replicates is plotted as error bars; two independent replicates were carried out (see Supplementary Figures S6, S7 for additional replicate). Spot dilutions for each culture confirms the turbidity measurements. Additional images for spot dilution experiments (*n* = 2) are given in Supplementary Figures S8, S9.

The differences in cellular toxicity of EcYoeB and AtYoeB are striking (Figure 2), given that canonical *in vitro* functions are conserved (Figure 3), as well as the limited differences in sequence and structure (Figure 1). We carried out superpositions of the AtYoeB toxin onto that previously determined for EcYoeB within the A-site of the ribosome (pre-cleavage state, PDB ID 6OXA, Pavelich et al., 2019). This highlighted a four amino acid helix in close proximity to the mRNA substrate (Figure 4A), particularly close to the first A in the co-crystallized AAU codon, which varies in sequence between the two toxins (Figure 1B, green box).

We reasoned this region may be responsible for the noted species-specific toxicity and carried out a helix-swapping experiment, replacing this four amino acid sequence in AtYoeB with that from EcYoeB and *vice versa*. When toxicity was again assessed using culture-based techniques, the wild type YoeB toxins displayed the expected toxicity only for their native hosts (Figures 4B,C). However, the chimeric version of AtYoeB (Figure 4B, “AtYoeB Ec helix”) greatly diminished the resulting turbidity of *E. coli* cultures, with some restored growth afforded to *A. tumefaciens* cultures. Similarly, chimeric EcYoeB (Figure 4B, “EcYoeB At helix”) was less toxic *E. coli* cells but was able to potently diminish *A. tumefaciens* turbidity. These cultures were utilized in a spot dilution assay, which recapitulated the effects observed by turbidity (Figures 4B,C).

### The Short Helix Sequence Is Highly Variable but Correlates With Proteobacterial Classes

**FIGURE 5:**
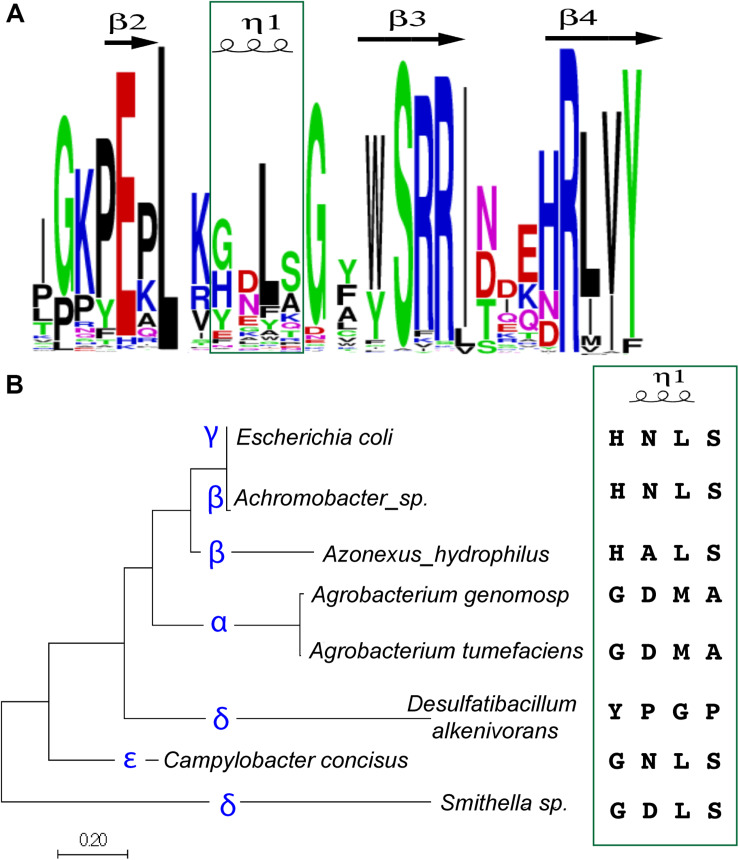
The sequence of the four amino-acid specificity-helix correlates with proteobacterial class. **(A)** A sequence logo was constructed based on alignments of YoeB sequences from the Pfam database. The specificity-helix is indicated in the green box. **(B)** Alignments of YoeB sequences were input to construct an evolutionary tree of sequence relationships; the specific class for the representative sequences is given (blue font). The specificity-helix sequence is listed for each of these classes. Note that for the beta class, the second position varies (Asn and Ala). Similarly, for the delta class, there is great variability throughout the four amino acids, with one sub-branch more similar to the epsilon class, while the other is fairly unique.

We questioned if the four amino-acid sequence of the short helix was actually species-specific, or if it were more broadly conserved. We examined the relationship between sequence variants within the Pfam 06769 family (El-Gebali et al., 2019) and discovered that variations in this region were limited to a few canonical signatures (Figure 5A, green box). The first amino acid can vary primarily between Gly, His, or Tyr, with Gly comprising the AtYoeB first amino acid and His found in the EcYoeB toxin. The second position is a polar residue, spanning Asp or Asn with some representation of Glu residues, consistent with the At and EcYoeB toxins, and each of these identities is appropriate for forming hydrogen bonds to the first base in the recognized codon. The third position highly favors a Leu, as found in EcYoeB, with Phe and Tyr both represented more prominently than the Met amino acid in AtYoeB. The final amino acid of this helix region seems to comprise a smaller polar or neutral amino acid, with Ser as found in EcYoeB being the most common followed by Ala, as found in AtYoeB, then Lys, Gly, and Thr.

Strikingly, when examined in a phylogenetic context these signatures specifically at the four amino acid helix partitioned relatively well to classes of proteobacteria (Figure 5B). The sequence found in *A. tumefaciens* is shared throughout the alpha class and is comprised of “Gly Asp Met Ala.” The sequence found within gamma proteobacteria, including *E. coli*, as well as the delta class is “His Asn(Ala) Leu Ser.” The delta and epsilon classes also seem mixed, with a consensus of “Gly(Tyr) Asp.Asn/Pro Leu(Gly) Ser(Pro)”. Based on the superposition within the ribosomal site, it seems that the first position can contribute to longer hydrogen bonding when a His, although clearly not as a Gly. They identification of a sub-class of delta proteobacteria with Tyr in this first position is fairly unique and expected to impact the fit within the ribosomal site, at least as based on the *E. coli* model. This unique sequence in the delta class is followed by a Pro, whereas all the other classes (including other delta proteobacteria) encode an amino acid to hydrogen bond with RNA bases. The third amino acid is hydrophobic, either Leu or Met, with this unique sequence again representing the outlier as Gly. The fourth and final amino acid in this helix is either Ser or Ala, with a Pro in the unique sub-sequence of delta proteobacteria. The conservation of specific variants within classes of proteobacteria is consistent with the proposed role of this four amino acid helix in species (or indeed, class) specificity.

## Discussion

The AtYoeB structure and sequence are highly similar to the well-characterized YoeB toxin from *E. coli* (Cherny et al., 2005; Kamada and Hanaoka, 2005; Zhang and Inouye, 2009; Chan et al., 2011; Feng et al., 2013; Pavelich et al., 2019), making the observed lack of toxicity of AtYoeB to *E. coli* cultures puzzling. As opposed to previously studied YoeB toxins, AtYoeB can be readily produced in *E. coli* in the absence of antitoxin, providing an ideal system to further explore YoeB activity. There are specific sequence changes at toxin-antitoxin interaction points, which would limit cross-reactivity of the antitoxins from different YoeB-YefM operons. The AtYoeB-YefM complex is a heterotetramer, and individual toxin and antitoxin polypeptides are dimeric. AtYoeB and AtYefM interact with canonically tight affinity in the high picomolar range. This high affinity can also be inferred by an increased melting transition point of approx. 20°C, while the AtYefM antitoxin does not contain sufficient folded structure to yield a hydrophobic core as needed to produce a signal in this assay.

Examination of sequence variants and available structures pointed to a four amino acid helix that varies in sequence and appears to make important contacts with the first base of the mRNA codon in the ribosome (Pavelich et al., 2019). Interestingly, of the published YoeB toxin characterizations, all have been toxic to *E. coli* regardless of their host bacteria. These have included a fungal endosymbiont with a specificity helix sequence similar to *E. coli* (Glu Ser Leu Ser), as well as Gram-positive bacterial species with similar sequences to EcYoeB (*Streptomyces*, Gly Asp Leu Ser; *S. aureus* YoeB-1, Ser Asn Leu Thr) and those with dissimilar sequences to EcYoeB (*Streptococcus* sp., Tyr Asp Tyr Gln; *S. aureus* YoeB-2, Pro Lys Try Leu) (Nieto et al., 2007; Yoshizumi et al., 2009; Nolle et al., 2013; Zheng et al., 2015; Salvioli di Fossalunga et al., 2017; Chan et al., 2018; Zhan et al., 2019). The four amino acid helix appears to interact with the first base of the codon selected for cleavage by YoeB interactions. EcYoeB appears to have some codon preferences, seemingly with a weak preference for adenine or guanine in the first position (Christensen et al., 2004; Pavelich et al., 2019). We carried out a series of experiments with helix-swapped versions and determined that this alone can alter toxicity. We also carried out a swap of just the polar amino acid (Asp versus Asn) and no reproducible impact was noted on toxicity (Supplementary Figures S6, S7), clearly indicating an impact of the entire helix. This four amino acid sequence, then, appears to impart at least a species-specific effect, although it is not clear if this arises due to different contacts with the mRNA substrate, or from other impacts upon interaction with ribosomes. We are also able to correlate the sequence of this helical region with different classes of proteobacteria, indicating that this mechanism of toxicity determination may be more broadly distributed. While we have not formally tested the link between class specificity and toxicity, the current work attests to a species-specific effect.

It remains possible that the efficiency of YoeB RNase activity varies between the Ec and AtYoeB toxins. However, this would not lead to the species-specific change in toxicity we noted unless it is also related to the interaction with the Ec or At ribosomes. There are few reports that address the similarity of the Ec and At ribosomes; they have been demonstrated to be similar in size and composition but to have differential sensitivity to plant-derived Ribosome-Inactivation Proteins (Knopf, 1977; Girbes et al., 1993). Additionally and highly relevant, At ribosomes differ from their Ec counterparts in having a strict requirement for a Shine-Dalgarno sequence to initiate translation (Golshani et al., 2002; Ahmad et al., 2014), and the YoeB toxins from Ec and *S. aureus* have both been demonstrated to impact translation initiation (Yoshizumi et al., 2009; Zhang and Inouye, 2009). We cannot exclude the possibility of cross-reactions of the YoeB toxins with the respective species-specific antitoxin, thereby altering the native conditional cooperativity observed for transcriptional regulation of the chromosomal copy, or of activating other TA systems (Overgaard et al., 2008; Page and Peti, 2016).

The current study utilized an *in vitro* cell-free system to quantify translational inhibition by GFP production, and for AtYoeB the resulting inhibition is around 50% complete at 240 nM. Previous studies with EcYoeB used a similar *in vitro* system but directly monitored RNA integrity rather than protein production; this demonstrated a complete loss of RNA substrate by 240 nM (Zhang and Inouye, 2009). It is also feasible that the differential toxicity between AtYoeB and EcYoeB is a result of codon preference in conjunction with a differential sensitivity of the two bacteria. Additional studies examining the specific RNA degradation patterns mediated by each toxin would reveal the likelihood of such preferences playing a role in the species (or class)-specific toxicity. Finally, while we cannot rule out an impact of the inserted sequence of “GSGS” in the AtYoeB toxin, but based on the superposition of our AtYoeB structure onto the ribosome-bound EcYoeB it seems unlikely to have an impact on catalytic efficiency.

The current study highlights that a lack of toxicity of toxins to *E. coli* cultures, a commonly utilized strategy in the identification of TA systems, may not be indicative of inactive toxins but instead of a species-specific toxin activity. This also opens the possibility of producing greater quantities of recombinant toxin proteins in other bacterial classes as a means to further study their *in vitro* properties.

## Author’s Note

Portions of this manuscript have been released as a pre-print on the bioRxiv server (https://doi.org/10.1101/795211, McGillick et al., 2019).

## Data Availability Statement

Atomic coordinates and structure factors for the reported crystal structure have been deposited with the Protein Data bank under accession number 6N90. Protein entries are available at the UniProt site as Atu2017, ID A9CID9 (AtYoeB), and Atu2018, ID Q7CY23 (AtYefM).

## Author Contributions

This study was conceptualized by JA and CB. TM collected *in vitro* translation data. JM assisted with the growth experiments. ER carried out the crystallographic study. CB carried out the DSF and MALS experiments. JA carried out the BLI experiments. The data analysis was performed by all authors. The manuscript was written by JA and CB. All authors edited and approved the final version and contributed to data collection.

## Conflict of Interest

The authors declare that the research was conducted in the absence of any commercial or financial relationships that could be construed as a potential conflict of interest.

## Footnotes

1 http://n2t.net/addgene:60733
