# Supplementary material for: Identifying a Molecular Mechanism That Imparts Species-Specific Toxicity to YoeB Toxins
Source: Front Microbiol. 2020 May 21;11:959. doi: 10.3389/fmicb.2020.00959 (PMC7256200; doi:10.3389/fmicb.2020.00959)
Supplement: Supplementary file 1 [file Data_Sheet_1.pdf]

Supplemental Materials

Identifying a molecular mechanism that imparts species-specific toxicity to YoeB toxins

Jessica R. Ames[1], Julia McGillick[1], Tamiko Murphy[1], Eswar Reddem[1], Christina R. Bourne[1]

[1]University of Oklahoma, Department of Chemistry and Biochemistry, Norman, OK, USA

*** Correspondence:**
Christina R. Bourne
cbourne@ou.edu

Present Address: Jessica R. Ames, Department of Chemistry, Maynooth University, Maynooth, County Kildare, Ireland
Present Address: Eswar Reddem, Zuckerman Institute, Columbia University, Columbia, NY, USA

Keywords: YoeB toxin, toxicity, *E. coli, A. tumefaciens,* ribosome-dependent mRNase, species-specific toxicity.

Table S1. Materials used in these studies.

**Plasmids**	
pET28	pBR322, Kan^R, T7 promoter, additional C-terminal GST fusion with cleavage site generated in house
pET15b	pBR322 origin, Amp^R, T7 promoter
pET-Duet	pBR322 origin, Amp^R, two T7 promoter sites
pSRK	pBBR1 origin, Kan^R, *lac* promoter<br>Kind gift from Dr. Sean Crosson, University of Michigan; originally published in (1)
**Bacterial strains**	
E. coli BL21 DE3 (NEB)	**Genotype:** B derivative, *fhuA2 [lon] ompT gal (λ DE3) [dcm] ΔhsdS*<br>*λ DE3 = λ sBamHIo ΔEcoRI-B int::(lacI::PlacUV5::T7 gene1) i21 Δnin5*
E. coli MG1655	**Genotype:** K-12 derivative (CGSC 7740), *F$^-$ λ$^-$ ilvG rfb-50 rph-1*<br>Kind gift from Dr. Tyrell Conway, Oklahoma State University
A. tumefaciens C58	**Genotype:** Wild-type strain, positive for pTiC58<br>Kind gift from Dr. Ben Holt III, University of Oklahoma
**Coding sequences within constructs**	
Agrobacterium tumefaciens YoeB<br>(in pET28)	**Toxin:** M K L V W T L S S W D D Y E F W Q R T D A R M V E K I N D L I R N A K R T P F A G L G K P E P L K G D M A G Y W S R R I T A E H R F V Y R V S G S G S E Q R L E V I Q C R F H Y Q<br>**C-terminal Fusion tag:** G S E F L E V L F Q G P M S P I L G Y W K I K G L V Q P T R L L L E Y L E E K Y E E H L Y E R D E G D K W R N K K F E L G L E F P N L P Y Y I D G D V K L T Q S M A I I R Y I A D K H N M L G G C P K E R A E I S M L E G A V L D I R Y G V S R I A Y S K D F E T L K V D F L S K L P E M L K M F E D R L C H K T Y L N G D H V T H P D F M L Y D A L D V V L Y M D P M C L D A F P K L V C F K K R I E A I P Q I D K Y L K S S K Y I A W P L Q G W Q A T F G G G D H P P K S D L E H H H H H H
Agrobacterium tumefaciens YoeB<br>(in pSRK)	**Toxin:** M K L V W T L S S W D D Y E F W Q R T D A R M V E K I N D L I R N A K R T P F A G L G K P E P L K G D M A G Y W S R R I T A E H R F V Y R V S G S G S E Q R L E V I Q C R F H Y Q
Agrobacterium tumefaciens YoeB Ec helix<br>(in pSRK)	**Toxin:** M K L V W T L S S W D D Y E F W Q R T D A R M V E K I N D L I R N A K R T P F A G L G K P E P L K **H N L S** G Y W S R R I T A E H R F V Y R V S G S G S E Q R L E V I Q C R F H Y Q
Agrobacterium tumefaciens YefM	**N-terminal Fusion Tag:** M G S S H H H H H H S Q D P N S<br>**Antitoxin:** M A N V R F T E F R Q N F A T H F D R V L E T R A P L L V T R

(in pET-Duet)	Q G K E A V V V L A E G E Y E S M Q E T L H L L S N P A N A S R L R A S M G E L E R G D T I E R D P T E E
Agrobacterium tumefaciens<br><br>YefM-YoeB<br><br>(in pET-Duet)	**MCS1, N-terminal Fusion Tag:** M G S S H H H H H H S Q D P N S<br><br>**MCS1, Antitoxin:** M A N V R F T E F R Q N F A T H F D R V L E T R A P L L V T R Q G K E A V V V L A E G E Y E S M Q E T L H L L S N P A N A S R L R A S M G E L E R G D T I E R D P T E E<br><br>**MCS2, N-terminal Fusion Tag:** M W S H P Q F E K L E V L F Q G P G G<br><br>**MCS2, Toxin:** M K L V W T L S S W D D Y E F W Q R T D A R M V E K I N D L I R N A K R T P F A G L G K P E P L K G D M A G Y W S R R I T A E H R F V Y R V S G S G S E Q R L E V I Q C R F H Y Q
Escherichia coli YoeB<br><br>(in pSRK)	**Toxin:** M K L I W S E E S W D D Y L Y W Q E T D K R I V K K I N E L I K D T R R T P F E G K G K P E P L K H N L S G F W S R R I T E E H R L V Y A V T D D S L L I A A C R Y H Y
Escherichia coli YoeB At helix<br><br>(in pSRK)	**Toxin:** M K L I W S E E S W D D Y L Y W Q E T D K R I V K K I N E L I K D T R R T P F E G K G K P E P L K **G D M A** G F W S R R I T E E H R L V Y A V T D D S L L I A A C R Y H Y
**Primers used for mutagenesis** (changed bases are listed in lowercase)	
AtYoeB with EcYoeB helix, Fwd	atggcaGTTTCTGGTCCCGACGCA
AtYoeB with EcYoeB helix, Rev	gtcaccTTTCAGGGGTTCTGGCTTC
EcYoeB with AtYoeB helix, Fwd	ttgtcaGGATATTGGTCTCGGCGG
EcYoeB with AtYoeB helix, Rev	attgtgCTTCAGAGGCTCCGGTTTC

Table S2. Crystallographic statistics for the structure determination of AtYoeB.

	6N90
**Data Collection**	
Resolution Range (Å)	38.9 – 1.755 (1.81 – 1.755)
Space Group	P 1 2_1 1
Unit Cell	*a*=30.9 Å *b*=77.7 Å *c*=36.4 Å
Total Reflections	16748
Completeness (%)	97.3 (98)
I / sigma (I)	2.61 (1.3)
Wilson B-factor	23.9
R-merge	0.08
**Refinement**	
R-factor (%)	21.8 (26.5)
R-free (%)	24.6 (34.1)
Number of Atoms	1591
**Model Quality**	
Num. amino acids / B factor ($Å^2$)	176 / 28.2
Num. water mols / B factor ($Å^2$)	123 / 28.3
Number of TEW / B factor ($Å^2$)	1 / 29.7
RMS Deviations, Bonds (Å)	0.01
RMS Deviations, Angles (°)	1.186
Ramachandran Favored (%)	93.3
Ramachandran Allowed (%)	6.7
Coordinate error	0.24
Clashscore	2.80

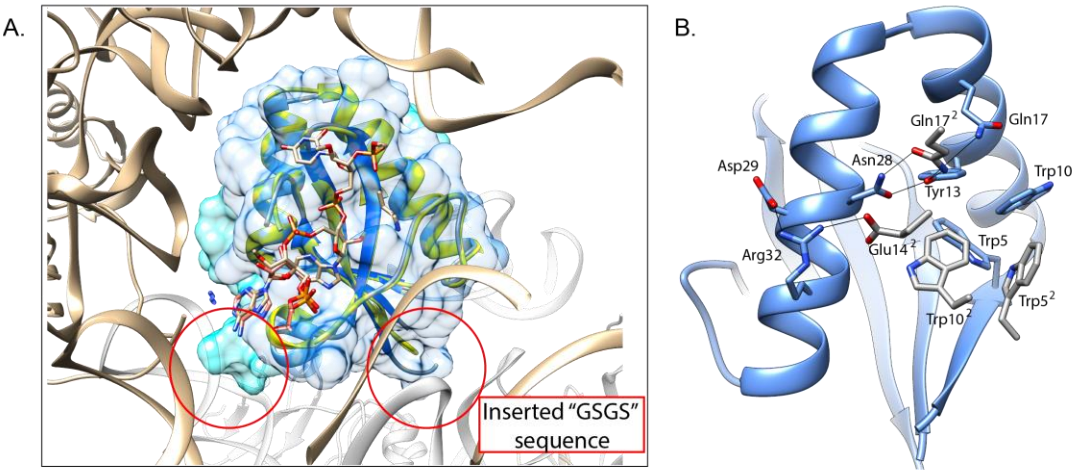

Figure S1. The AtYoeB dimer has an inserted sequence relative to EcYoeB, as well as amino acid differences at the dimer interface. A. The AtYoeB structure (blue ribbons and surface, PDB 6N90) was superposed onto the previously published EcYoeB toxin (yellow ribbons) within the *E. coli* ribosomal A-site (tan ribbons, target mRNA as sticks, PDB 6OXA, 6OTR) (2). This exercise indicates that the inserted “GSGS” sequence (within the red circles) in AtYoeB is not expected to make any contacts within the functional complex for either monomer. **B.** The dimer interface of AtYoeB contains the canonical Trp stack, comprised of Trp5 and Trp10, in addition to numerous stabilizing polar interactions. The identity at position 14 in AtYoeB is polar and contributes additional electrostatic interactions as compared to the EcYoeB. Changes at position 18 and 32 are more conservative and maintain the electrostatic interactions. Note that the second molecule is obscured for a clearer view; further, only one set of the symmetrical interactions are shown.

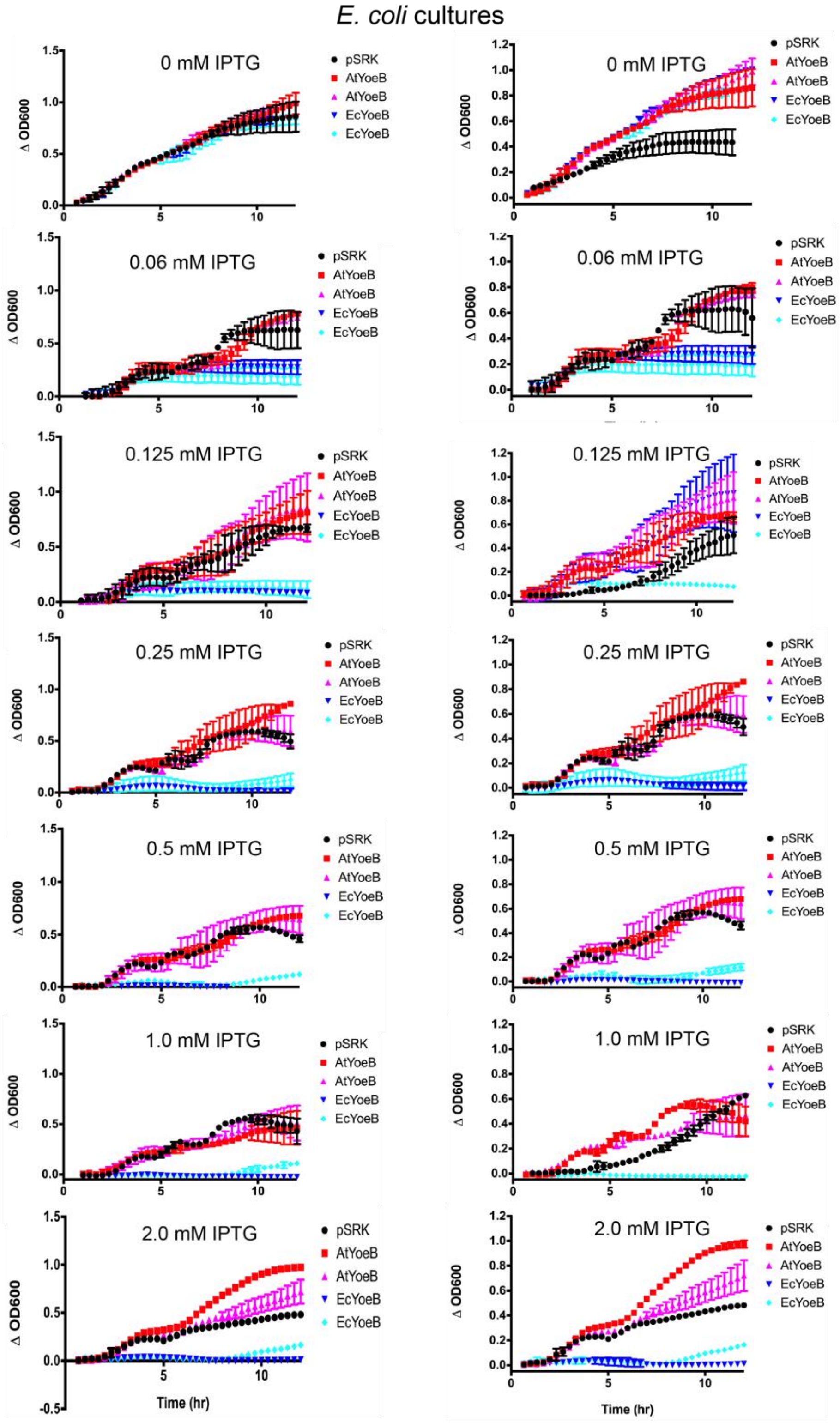

Figure S2. EcYoeB, but not AtYoeB, is toxic when expressed in *E. coli* cultures. Two sequence-verified clones of AtYoeB (red, magenta) and EcYoeB (blue, cyan) were compared to the pSRK vector with no insert (black) to assess the impact on growth turbidity. EcYoeB appears to complete block cell replication at inductant concentrations of 0.125 mM IPTG and higher, while AtYoeB does not impact turbidity to at least 2 mM IPTG.

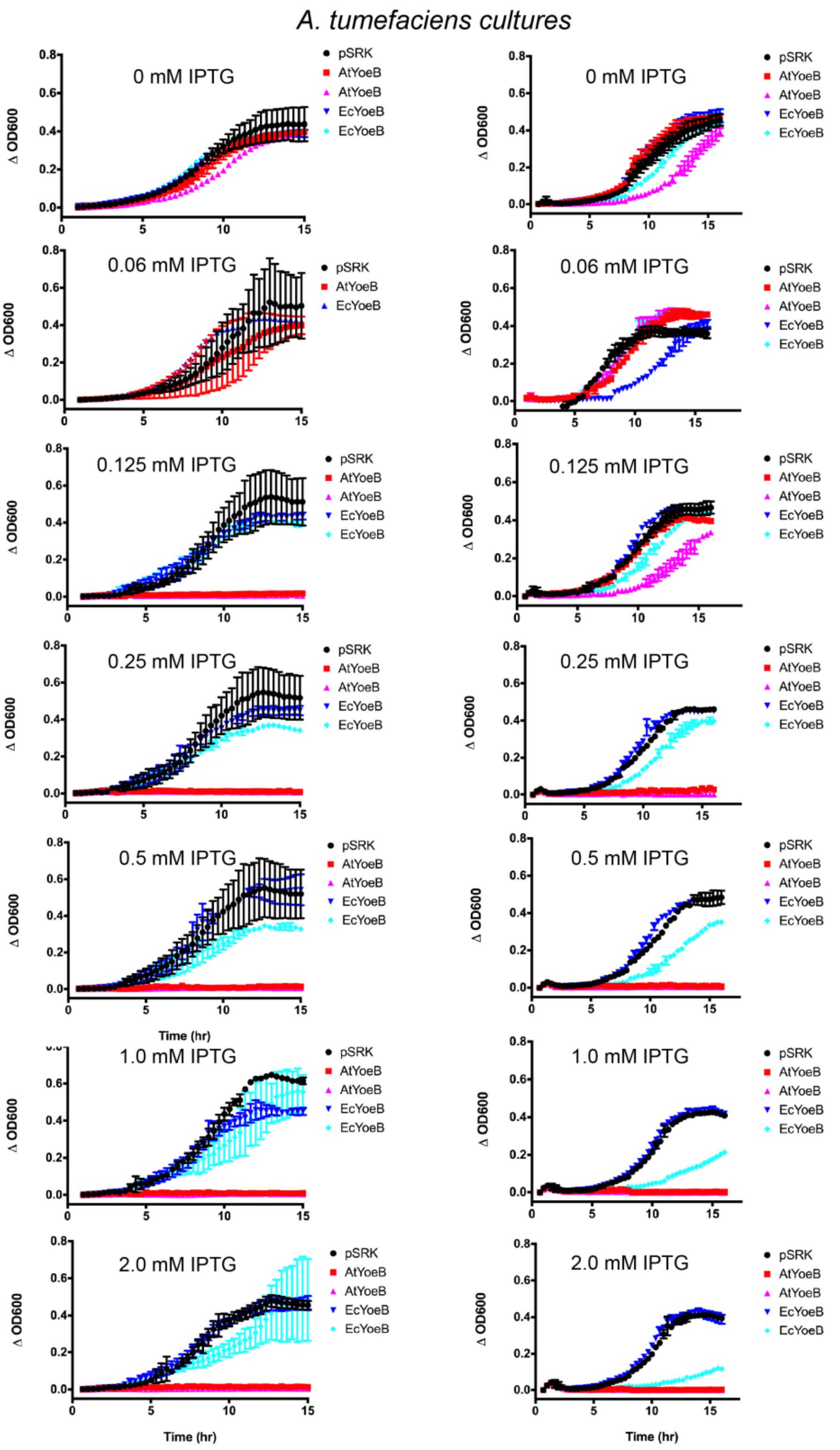

Figure S3. AtYoeB, but not EcYoeB, is toxic when expressed in *A. tumefaciens* cultures. Two sequence-verified clones of AtYoeB (red, magenta) and EcYoeB (blue, cyan) were compared to the pSRK vector with no insert (black) to assess the impact on growth turbidity. AtYoeB appears to effect cell replication at inductant concentrations of 0.125 mM IPTG and higher, while EcYoeB does not impact turbidity to at least 1 mM IPTG.

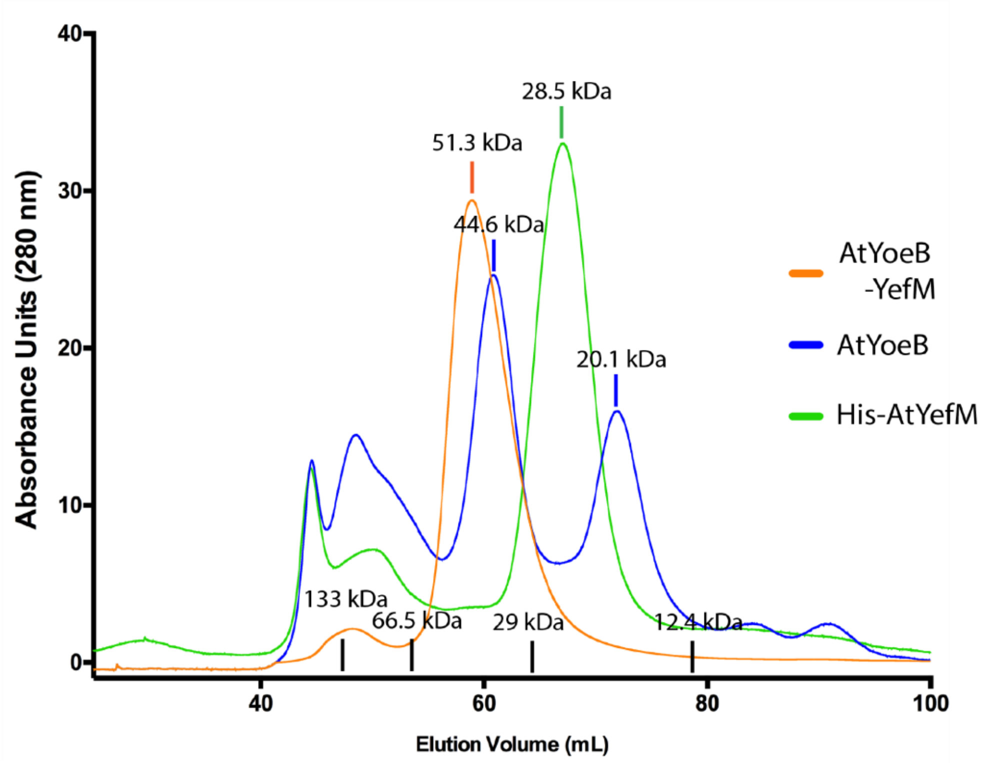

Elution volumes:

78.7	12.400 kDa	(Cytochrome C)
67.4	29.000 kDa	(Carbonic Anhydrase)
53.1	66.500 kDa	(BSA monomer)
47.3	133.000 kDa	(BSA dimer)
58.9	51.331 kDa	AtDE4
67.1	28.476 kDa	AtD4
60.9	44.652 kDa	GST dimer
71.9	20.126 kDa	AtE4

Figure S4. Size exclusion analysis of AtYoeB after cleavage of its GST-His fusion affinity tag, of AtYefM with its His affinity tag, and of the co-expressed AtYoeB-YefM complex (containing a His tag on the YefM and a Strep tag on the YoeB). Each sample was first purified by Ni-NTA, concentrated, and directly applied to a Sephadex S-75 column. Note that the co-expressed sample does not contain additional peaks that would correspond to any other oligomeric species aside from the heterotetramer. Standard sized proteins used to calibrate the column are listed, as are the calculated molecular masses based on elution position.

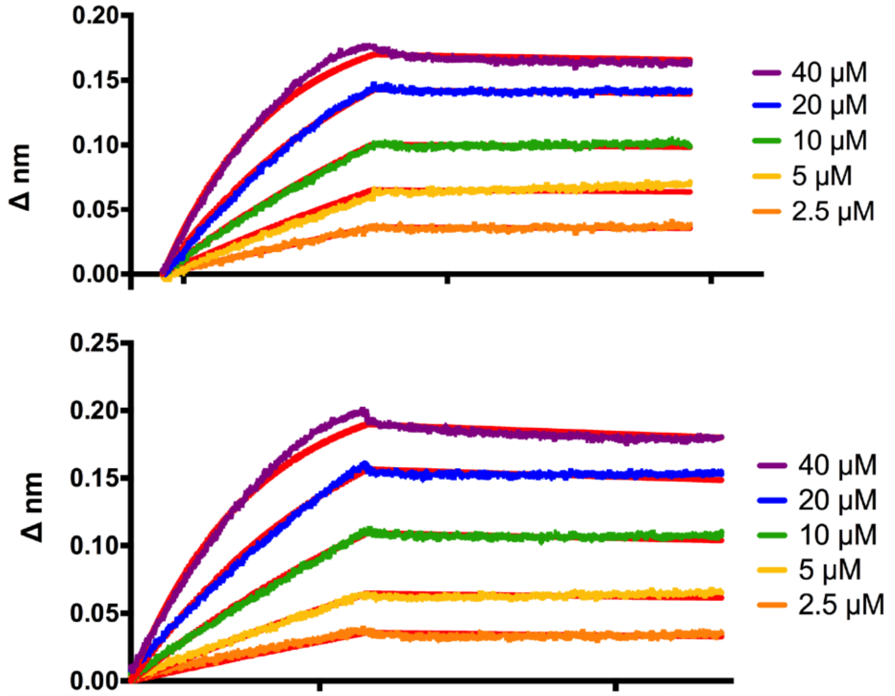

K_D (M)	k_{on} (1/Ms)	k_{diss} (1/s)	Chi^2	R^2
$3.22 \pm 0.20 \times 10^{-10}$	$1.19 \pm 0.008 \times 10^{5}$	$3.84 \pm 0.23 \times 10^{-5}$	0.043	0.997
$8.91 \pm 0.25 \times 10^{-10}$	$6.95 \pm 0.05 \times 10^{4}$	$6.19 \pm 0.17 \times 10^{-5}$	0.031	0.999
$7.45 \pm 0.21 \times 10^{-10}$	$1.19 \pm 0.008 \times 10^{5}$	$8.86 \pm 0.24 \times 10^{-5}$	0.041	0.997
Average Values				
$6.53 \pm 3 \times 10^{-10}$	$9.43 \pm 3.5 \times 10^{4}$	$3.74 \pm 2.5 \times 10^{-5}$		

Figure S5. BLI data used to calculate an interaction strength between AtYoeB and AtYefM of 653 pM. The AtYefM antitoxin was captured by an N-terminal 6× affinity tag to NiNTA pins, and titrations of AtYoeB toxin (affinity tag cleaved) were incubated with captured antitoxin. The data were fit to a 1:1 binding model; resulting values for rates of association and dissociation, the calculated equilibrium binding constant, and the error estimates from the fit are given.

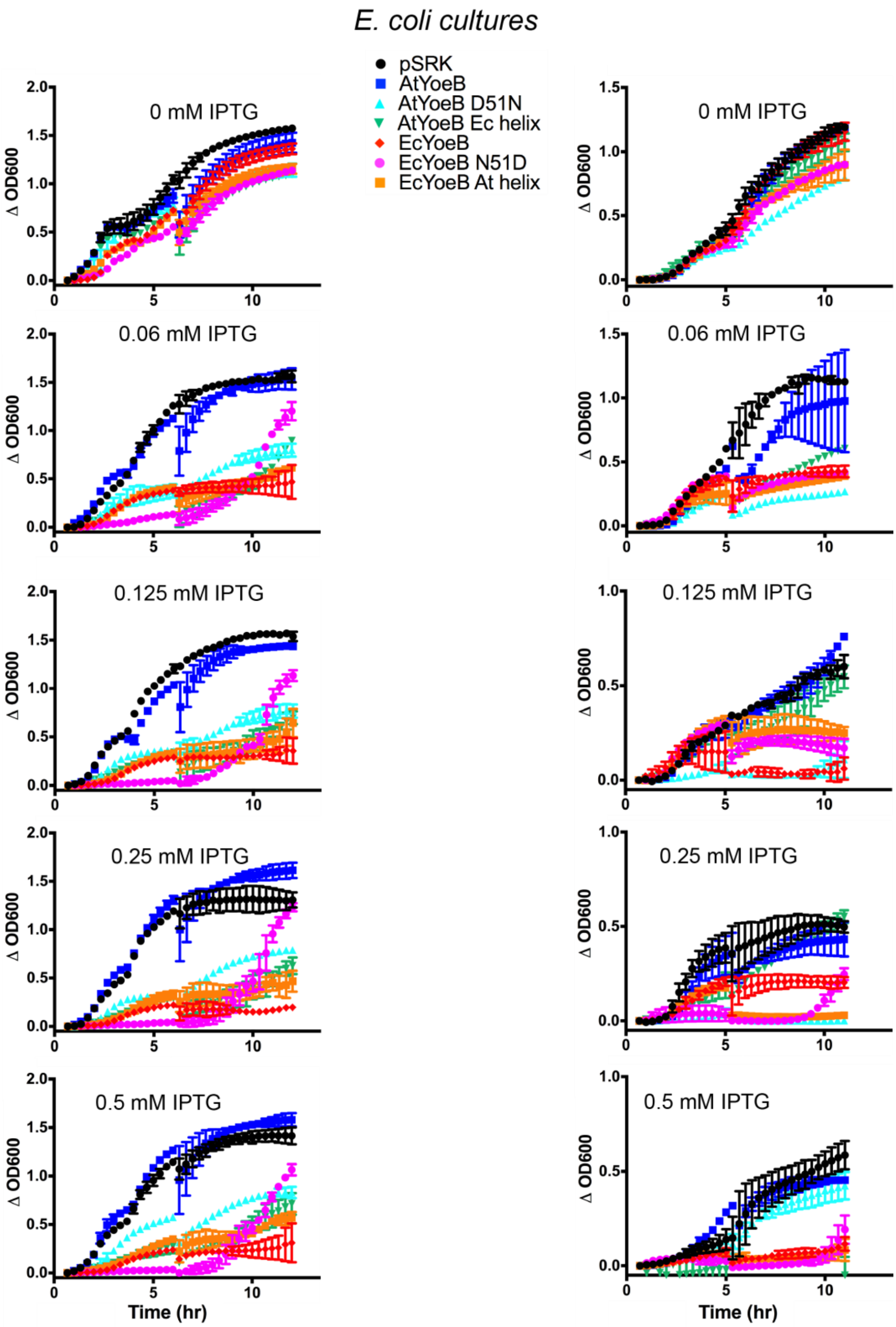

Figure S6. EcYoeB chimeric for the AtYoeB helix (orange) is less toxic to *E. coli* cultures but more toxic to *A. tumefaciens* cultures. Wild-type EcYoeB (red) maintains toxicity to its native host, as previously measured, while AtYoeB is not toxic to *E. coli* (blue). However, swapping the four amino acid sequence (wild type Ec encodes HNLS, wild type At encodes GDMA) alters the resulting toxicity.

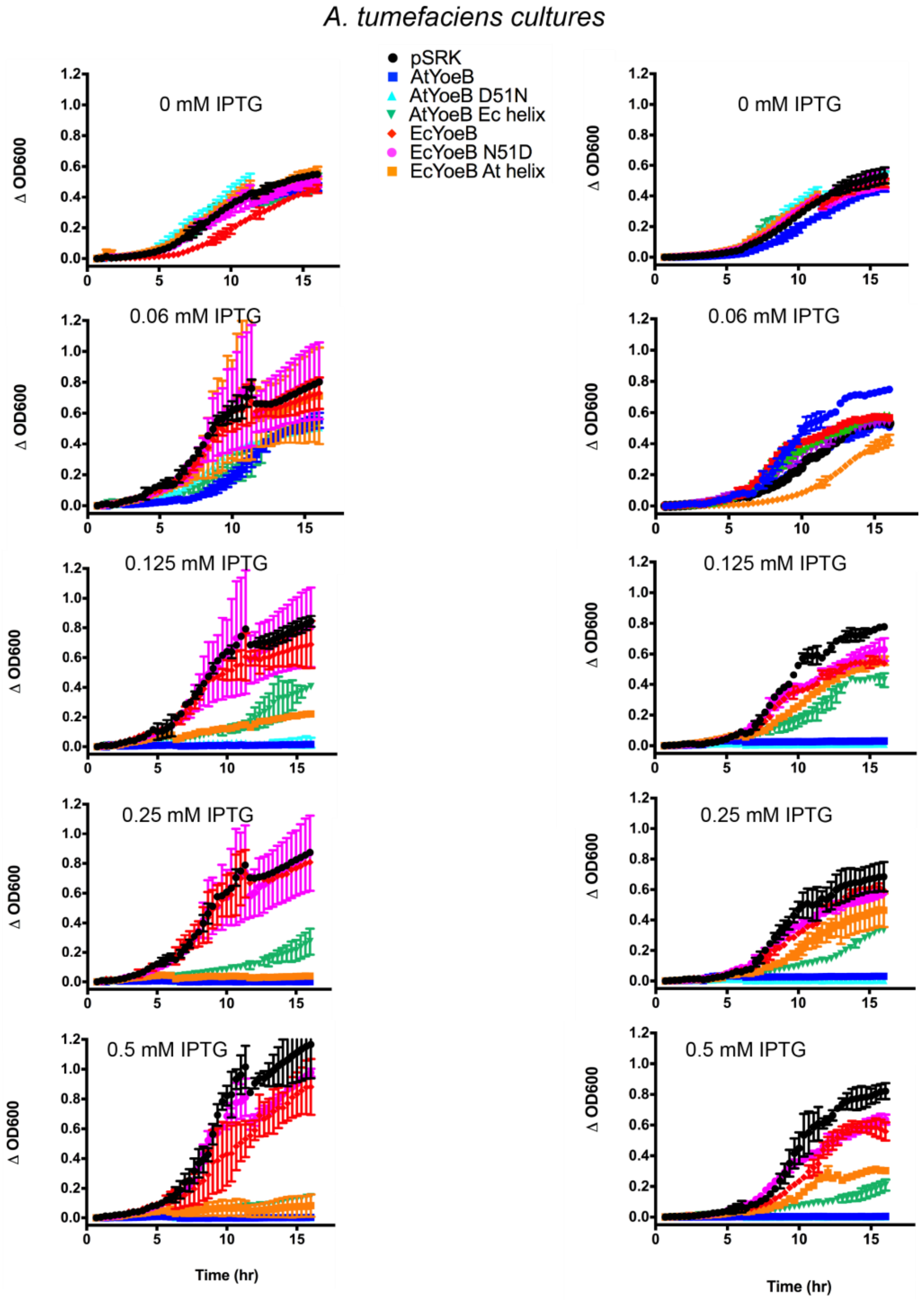

Figure S7. AtYoeB chimeric for the EcYoeB helix (green) is less toxic to *A. tumefaciens* cultures but more toxic to *E. coli* cultures. Wild-type AtYoeB (blue) maintains toxicity to its native host, as previously measured, while EcYoeB is not toxic to *A. tumefaciens* (red). However, swapping the four amino acid sequence (wild type Ec encodes HNLS, wild type At encodes GDMA) alters the resulting toxicity.

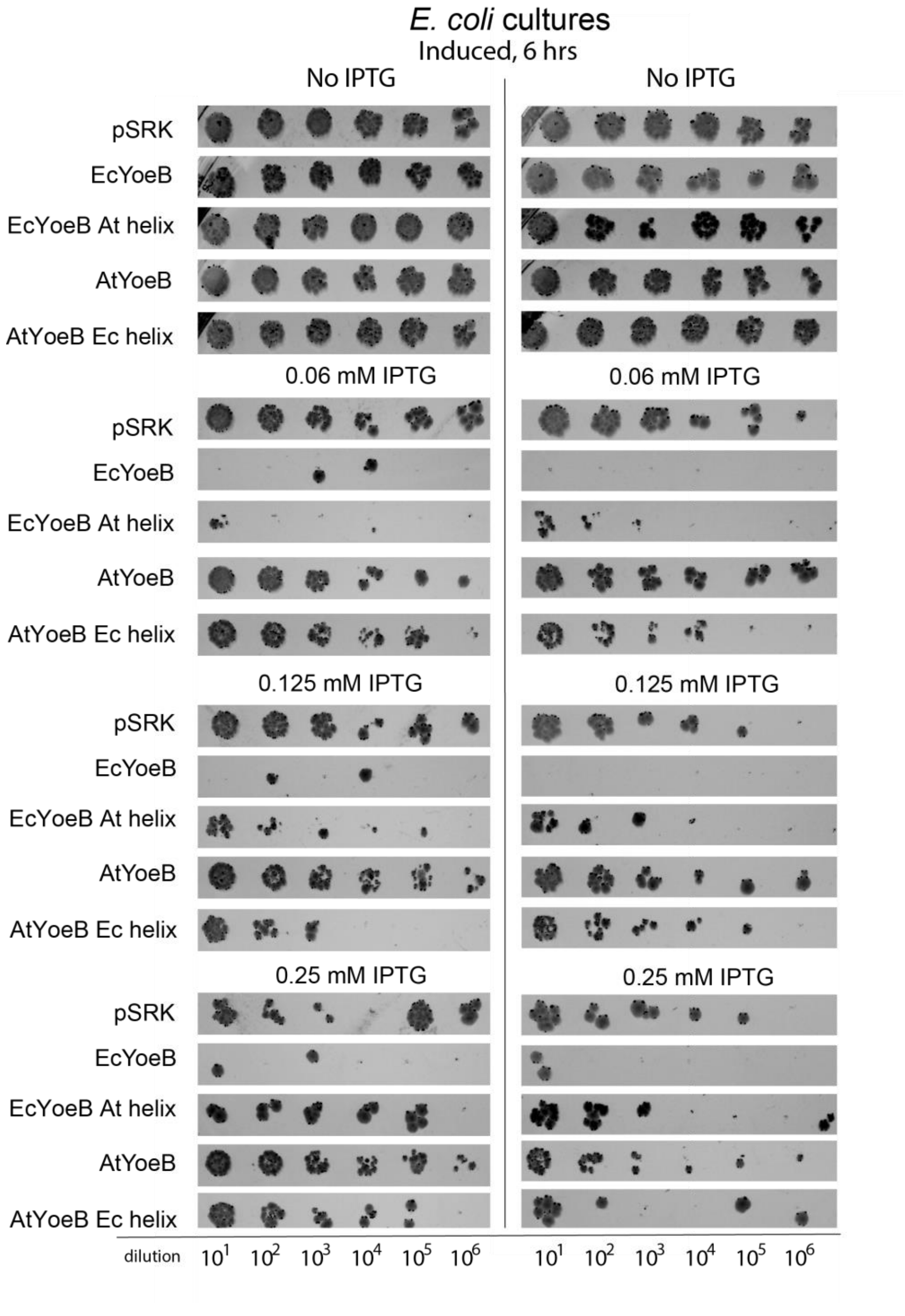

Figure S8. Spot dilution assays of *E. coli* cultures verify the impacts on toxicity noted in turbidity measurements.

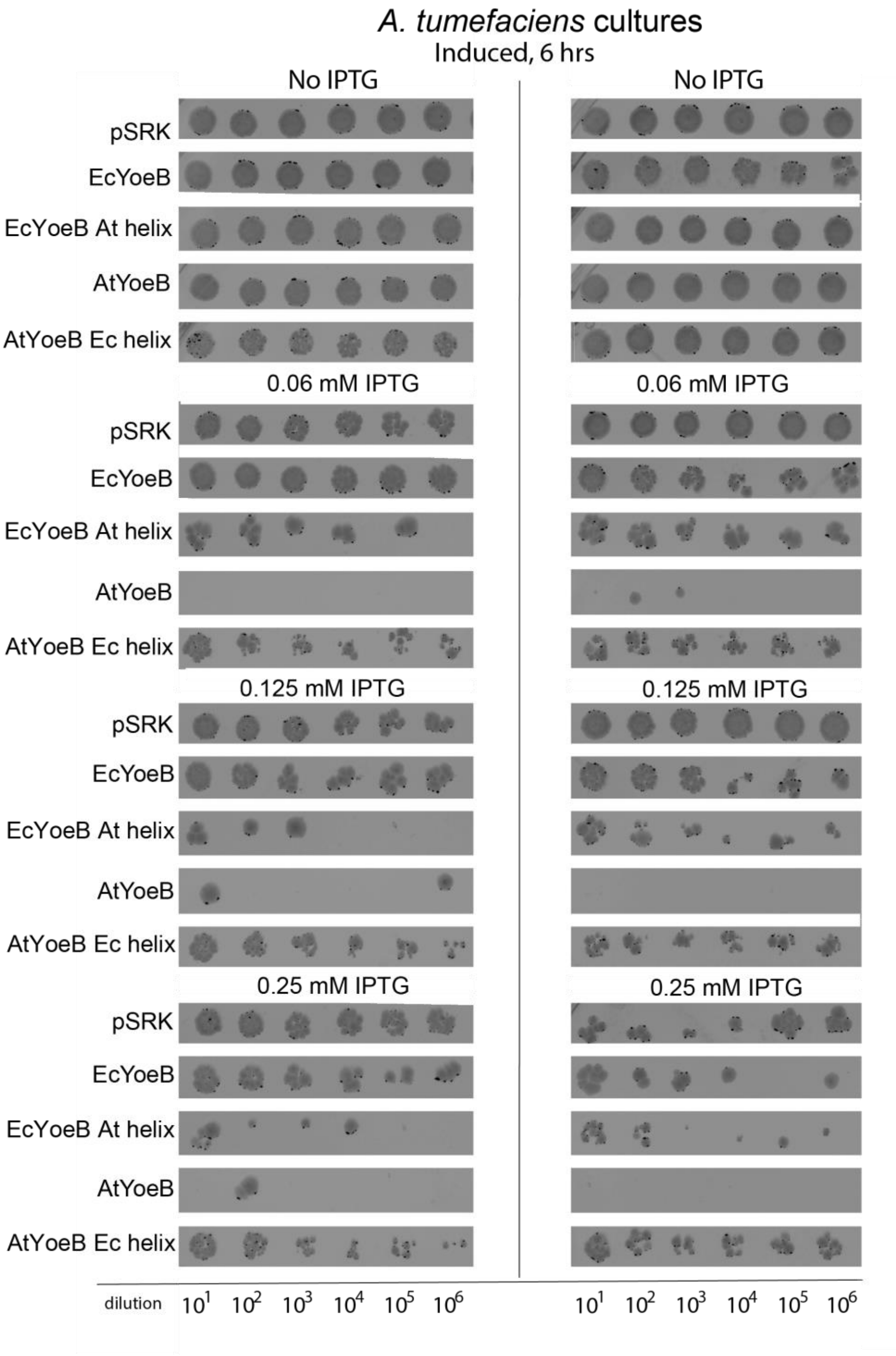

Figure S9. Spot dilution assays of *A. tumefaciens* cultures verify the impacts on toxicity noted in turbidity measurements.

1. Khan, S.R., Gaines, J., Roop, R.M., 2nd and Farrand, S.K. (2008) Broad-host-range expression vectors with tightly regulated promoters and their use to examine the influence of TraR and TraM expression on Ti plasmid quorum sensing. *Applied and Environmental Microbiology*, **74**, 5053-5062.
2. Pavelich, I.J., Maehigashi, T., Hoffer, E.D., Ruangprasert, A., Miles, S.J. and Dunham, C.M. (2019) Monomeric YoeB toxin retains RNase activity but adopts an obligate dimeric form for thermal stability. *Nucleic Acids Research*, **47**, 10400-10413.
